# Probing Homogeneous Catalysts and Precatalysts in Solution by Exchange-Mediated Overhauser Dynamic Nuclear Polarization NMR

**DOI:** 10.1021/jacs.4c01570

**Published:** 2024-04-30

**Authors:** Yu Rao, Federico De Biasi, Ran Wei, Christophe Copéret, Lyndon Emsley

**Affiliations:** † Institut des Sciences et Ingénierie Chimiques, Ecole Polytechnique Fédérale de Lausanne (EPFL), CH-1015 Lausanne, Switzerland; ‡ Department of Chemistry and Applied Biosciences, ETH Zürich, CH-8093 Zürich, Switzerland

## Abstract

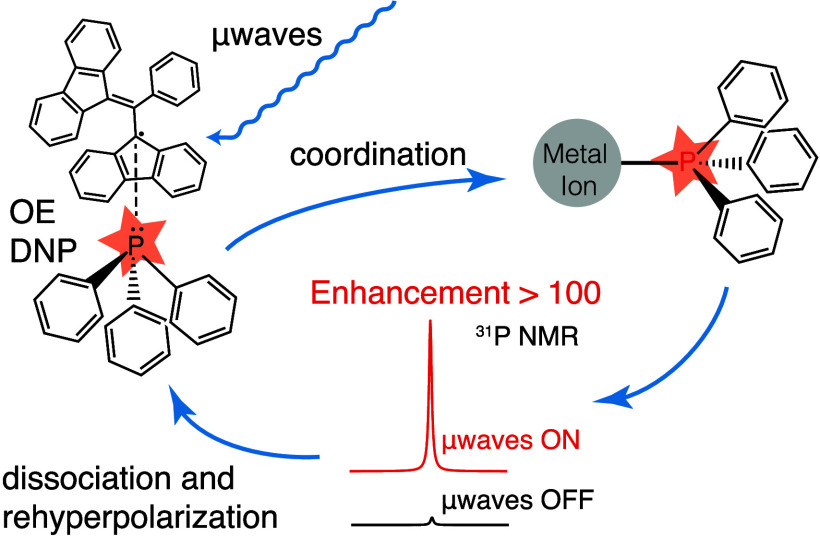

Triphenylphosphine (PPh_3_) is a ubiquitous ligand in organometallic chemistry that has been shown to give enhanced ^31^P NMR signals at high magnetic field via a scalar-dominated Overhauser effect dynamic nuclear polarization (OE DNP). However, PPh_3_ can only be polarized via DNP in the free form, while the coordinated form is DNP-inactive. Here, we demonstrate the possibility of enhancing the ^31^P NMR signals of coordinated PPh_3_ in metal complexes in solution at room temperature by combining Overhauser effect DNP and chemical exchange between the free and coordinated PPh_3_ forms. With this method, we successfully obtain ^31^P DNP enhancements of up to 2 orders of magnitude for the PPh_3_ ligands in Rh(I), Ru(II), Pd(II), and Pt(II) complexes, and we show that the DNP enhancements can be used to determine the activation energy of the ligand exchange reaction.

## Introduction

Tertiary phosphines correspond to a ubiquitous class of spectator ligands in organometallic chemistry and homogeneous catalysis.^1−3^ Phosphines are typically used to stabilize metal complexes and reaction intermediates in catalysis. In particular, triphenylphosphine (PPh_3_) is a frequently used tertiary phosphine owing to its high air stability, accessibility, and low toxicity.^4^ Notably, PPh_3_ plays a pivotal role in a series of textbook transition-metal complexes such as [Pd(PPh_3_)_4_], [Rh(PPh_3_)_3_Cl] (Wilkinson’s catalyst), [RhH(CO)(PPh_3_)_3_], and [Ir(CO)(PPh_3_)_2_Cl] (Vaska’s complex), some of them being used in now classical transition-metal-catalyzed organic transformations such as the Heck reaction^5^ (Negishi, Stille Suzuki, etc.), cross-coupling reactions,^6^ as well as the hydrogenation,^7,8^ hydroformylation,^9^ and hydrosilylation^10^ of olefins to name a few.

While this chemistry is well established, characterization of such compounds in diluted solution relevant to catalysis and necessary to study reaction mechanisms remains challenging. Indeed, ^31^P NMR is a powerful tool to investigate the mechanism of many important catalytic reactions,^11,12^ since the chemical shift window and the *J* couplings of ^31^P are very informative regarding metal complexation. Even if ^31^P is one of the most sensitive nuclei for NMR due to the favorable spin properties and high abundance, the intrinsic low sensitivity of NMR poses severe challenges to the observation of ^31^P spectra in many cases, particularly when the detection of species at low concentrations is required.

To address the sensitivity limitation, over the past few decades, hyperpolarization methods have gained considerable interest as they provide a versatile platform to enhance NMR signals beyond the thermal limit, boosting the sensitivity of NMR experiments by at least 2 orders of magnitude in most cases, when they can be applied.^13,14^ Nevertheless, the majority of liquid-state hyperpolarization protocols yield transient, nonrenewable hyperpolarized signals.^15,16^ Strong NMR signals are then detected in a single-shot fashion, or by using a series of small flip-angle excitation pulses. Examples of such techniques are dissolution dynamic nuclear polarization (dDNP),^17^ parahydrogen induced polarization (PHIP)^18^ and signal amplification by reversible exchange (SABRE)^19^ (though we note that SABRE can provide continuous and replenishable hyperpolarization if dedicated hardware is used). Recently, a novel method consisting of the rapid dissolution of optically polarized naphthalene crystals was also shown to generate transient nuclear hyperpolarization in the liquid state.^20^

On the contrary, Overhauser effect dynamic nuclear polarization (OE DNP) can generate steady-state nuclear hyperpolarization in solutions, as long as the electron spin transition of a suitable paramagnetic species is continuously saturated by microwave irradiation.^21,22^ In OE DNP, the polarization transfer from the paramagnetic species—the polarizing agent—to the nuclear spin of interest is mediated by electron–nuclear cross-relaxation.^23^ OE DNP can be very efficient at low magnetic fields (<1 T),^24^ but typically, the hyperfine interaction responsible for the cross-relaxation term is dominated by the electron–nuclear dipolar coupling, and this leads to a substantial reduction in the polarization transfer efficiency at high magnetic fields such that only small enhancements can be obtained at 9.4 T in most cases.^25^ However, previous studies have shown that for ^13^C and ^31^P nuclei in specific chemical environments, the hyperfine interaction with the corresponding polarizing agent can be scalar-dominated.^26^ In these cases, OE DNP enhancements of up to 3 orders of magnitude have been observed at high magnetic fields (≥9.4 T).^27,28^ Most importantly, PPh_3_ has previously been shown to provide OE DNP enhancements (ε), defined as the ratio of the signal integral detected with and without microwave irradiation (ε = *I*_z,on_/*I*_z,off_), of up to 150 at magnetic fields from 5 to 14.1 T using NMR probes with nonresonant microwave geometries.^26,29,30^

In this light, it would be of particularly great interest if the NMR signals of coordinated PPh_3_ ligands in organometallic catalysts could be also enhanced. However, the outstanding scalar OE DNP performance observed for free PPh_3_ is absent in its coordinated form. Indeed, the phosphorus lone pair plays a crucial role in the scalar-dominated hyperfine interaction with the polarizing agent. Due to the loss of this lone pair upon coordination to a metal site,^31^ no scalar OE DNP effects have been observed in PPh_3_-bound complexes thus far.^31−35^

**Scheme 1:**
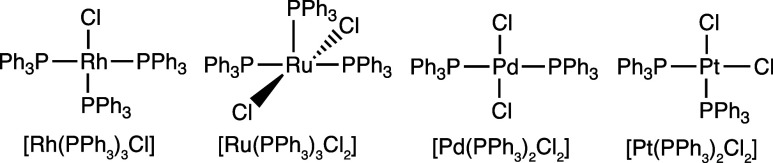
Chemical Structures and Formulae of the Metal Complexes Used in This Work

Here, we demonstrate an efficient approach to obtain DNP-enhanced ^31^P NMR spectra of coordinated PPh_3_ ligands based on chemical exchange between coordinated PPh_3_ and hyperpolarized free PPh_3_. We apply the proposed method to a series of prototypical transition-metal complexes used in homogeneous catalysis, namely, [Rh(PPh_3_)_3_Cl] (the Wilkinson catalyst), [Ru(PPh_3_)_3_Cl_2_], [Pd(PPh_3_)_2_Cl_2_], and [Pt(PPh_3_)_2_Cl_2_] (Scheme 1). Using a commercial Bruker 9.4 T magic angle spinning (MAS) DNP probe, we obtained ^31^P enhancements on the metal-coordinated PPh_3_ ligands in the catalysts of over a factor 100 and were able to observe coordinated ligand signals at submillimolar concentrations.

## Results and Discussion

**Scheme 2:**
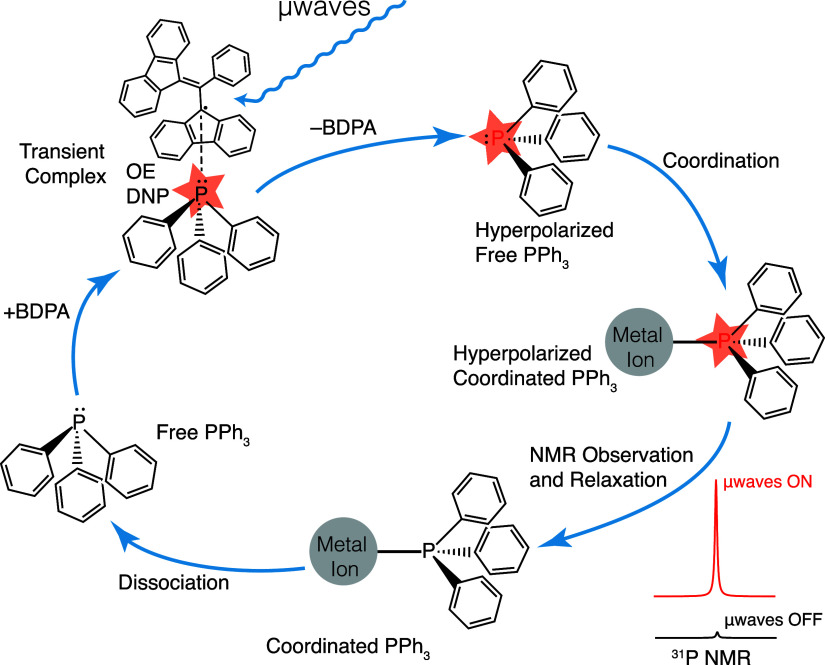
Schematic Representation of the Chemical Exchange Relayed Polarization Transfer Pathway Used to Enhance NMR Signals from PPh_3_ Ligands in Metal Complexes

Free PPh_3_ is readily hyperpolarized by OE DNP through a transient scalar hyperfine interaction between the unpaired electron in the radical center of (α,γ-bisdiphenylene-β-phenylallyl (BDPA), Scheme 2) and the ^31^P nuclear spin that is mediated by the lone pair of the PPh_3_. However, upon coordination of phosphorus to the metal center, the lone pair is no longer available to interact with the BDPA, obliterating the effect. Nevertheless, chemical exchange has been shown to enable polarization transfer from one species to another and can thus be exploited to enhance NMR signals. In fact, saturation transfer has been implemented in numerous examples in order to probe exchanging species,^36−38^ and Scheme 2 illustrates the polarization transfer pathway we propose to use to enhance the PPh_3_ ligand signals in transition-metal complexes. Indeed, reversible exchange between free and coordinated PPh_3_ is well known in solution; it is in fact a key elementary step in catalysis to generate reactive low-valent active species.^39^ Therefore, in principle, the chemical exchange can be exploited to allow hyperpolarized free PPh_3_ to back coordinate the metal center, leading to hyperpolarized coordinated PPh_3_. In order for this to occur, the exchange rate must be faster than the ^31^P longitudinal relaxation rate (1/*T*_1_) of free PPh_3_, otherwise, the hyperpolarization generated on the free ligands would relax before an exchange event takes place, on average.^40^ On the other hand, the exchange rate must not exceed the chemical shift difference between free and coordinated PPh_3_ (slow chemical exchange regime) so that a distinct DNP-enhanced ^31^P signal can be detected for the coordinated PPh_3_. To fulfill these requirements, we propose to use an excess of free PPh_3_, which is a common condition used for many catalytic reactions, such as hydroformylation,^41^ and to vary the temperature to modulate the exchange rates and improve hyperpolarization transfer efficiency.

To demonstrate the utility of this strategy, we applied the protocol on [Rh(PPh_3_)_3_Cl] (Wilkinson’s catalyst) and three other complexes with different metal centers, Ru(II), Pd(II), and Pt(II), as shown in Scheme 1.

**Figure 1:**
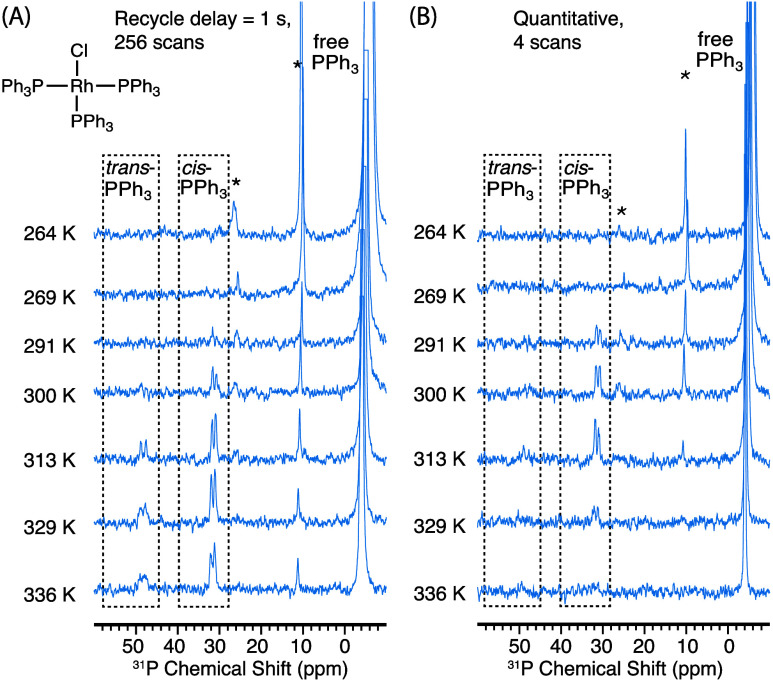
^31^P NMR spectra of a saturated solution of [Rh(PPh_3_)_3_Cl] (Wilkinson’s catalyst) in benzene-*d*_6_ with 10 mM BDPA used as the PA, and the addition of excess PPh_3_ (100 mM). All NMR experiments were performed at 9.4 T with continuous-wave microwave irradiation at different sample temperatures with (A) recycle delay of 1 s, 256 scans; (B) recycle delay >5 × *T*_1_ of free PPh_3_ (values shown in Table S3), 4 scans. The spinning sidebands are labeled with asterisks and are due to imperfect shimming. The resonances due to coordinated PPh_3_ are labeled with *trans-*PPh_3_ and *cis-*PPh_3_.

Experiments on a saturated solution (∼2 mM) of [Rh(PPh_3_)_3_Cl] (Wilkinson’s catalyst) with 10 mM BDPA in benzene-*d*_*6*_ in the presence of an excess amount of PPh_3_ as the polarizable medium were carried out at 9.4 T. Further details on the DNP system used can be found in the Experimental Section. To compensate for the microwave-induced heating, the sample is actively cooled by a cold nitrogen gas flow. The sample temperature is estimated from the ^31^P chemical shift of free PPh_3_ and is adjusted using the temperature and flow rate of the cooling gas, as described in the Experimental Section. As shown in Figure 1A, at temperatures below 290 K, we only observe the DNP-enhanced ^31^P signal of free PPh_3_ (δ ≈ −5 ppm), whereas the signal of coordinated PPh_3_ cannot be detected, consistent with the very slow rate of chemical exchange at this temperature. As the temperature increases (>300 K), we clearly observe enhanced metal-coordinated PPh_3_ signals, as polarization transfer is facilitated by the accelerated exchange between the hyperpolarized free PPh_3_ and coordinated PPh_3_. This is proven by the appearance of two peaks at δ = 32 and 48 ppm, which are attributed to the coordinated PPh_3_ ligands in *cis* and *trans* positions relative to the chlorine atom, respectively.^42^ Signal intensity of the bound ligands continues to increase as the temperature increases, reaching a plateau near the boiling point of benzene (336 K) and yielding a ^31^P signal enhancement of approximately a factor of 16 at 329 K (Figure S4A).

Both the coordinated PPh_3_ peaks in Figure 1 appear as doublets due to the scalar coupling with 100% abundant ^103^Rh, with coupling constants *J*_Rh–P_ of 142 and 189 Hz for *cis*- and *trans*-PPh_3_, respectively. The observation of these resolved couplings confirms that the ligand exchange rate is slower than *J*_Rh–P_. (We note that we do not observe a resolved ^31^P–^31^P *J* coupling, but that the line widths in the MAS DNP setup used here are on the order of the expected value of the coupling (∼40 Hz)).

Figure 1B shows the DNP-enhanced ^31^P NMR spectra acquired under quantitative conditions (i.e., recycle delay >5 × *T*_1_). In these experiments, the enhancements observed on coordinated PPh_3_ show an initial increase with temperature, followed by a rapid decrease after 313 K. At 313 K, a maximum enhancement of a factor 50 was obtained on coordinated PPh_3_ (Figure S4B). The decrease in enhancement at high temperatures is attributed to a loss of polarization, which is a side effect of ligand exchange and is discussed further below.

**Figure 2:**
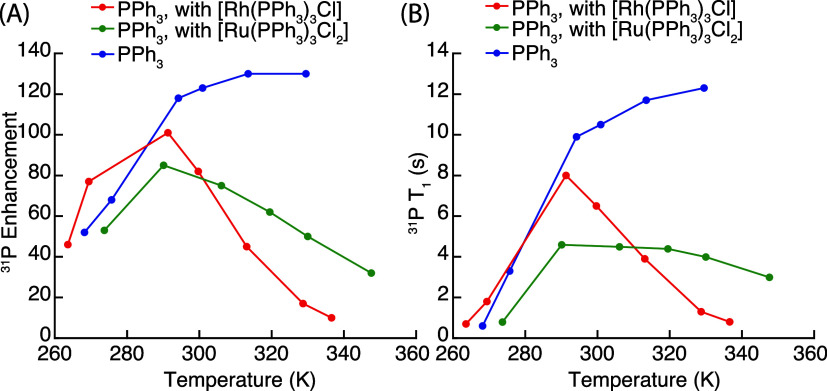
(A) ^31^P DNP enhancement under quantitative NMR conditions and (B) the apparent *T*_1_ measured for the free PPh_3_ signal (100 mM) without metal complexes (blue), with [Rh(PPh_3_)_3_Cl] (red) and [Ru(PPh_3_)_3_Cl_2_] (green), respectively, under microwave irradiation at 9.4 T. In 10 mM BDPA benzene-*d*_6_ solutions.

To understand the temperature-dependent behavior of the coordinated PPh_3_ enhancement, we measured the enhancement of free PPh_3_ under quantitative conditions and its *T*_1_, in the absence of metal complexes (Figure 2, blue traces) and in the presence of [Rh(PPh_3_)_3_Cl] (Figure 2, red traces). In the absence of complexes, both *T*_1_ and the enhancement display a monotonic increase with temperature, consistent with previous studies.^27^ In contrast, in the presence of [Rh(PPh_3_)_3_Cl], the apparent *T*_1_ of free PPh_3_ and the DNP enhancement under quantitative conditions initially increase with temperature, but then decrease at temperatures above 291 K (Figure 2, red). A rationale for this observation is the shorter *T*_1_ for the coordinated form of PPh_3_ compared to that of free PPh_3_. As shown in Table S7, the *T*_1_ of the coordinated PPh_3_ is shorter than 1 s at room temperature. The increased exchange rate at higher temperatures thus leads to a decrease in the apparent *T*_1_ of free PPh_3_. Furthermore, since PPh_3_ can only be hyperpolarized in the free form, as the exchange rates increase, the coordinated PPh_3_ acts as an efficient relaxation sink and makes it more difficult to accumulate polarization. If the relaxation sink effect outweighs the intrinsic positive temperature dependence of OE DNP, the enhancement will decay, as observed above 291 K in Figure 2A.

In light of the behavior of free PPh_3_, the temperature dependence of the coordinated PPh_3_ enhancement shown in Figure 1 can be explained. Since the polarization of the coordinated PPh_3_ is predominantly ascribed to chemical exchange with hyperpolarized free PPh_3_, the overall enhancement initially increases with faster exchange rates. The enhancement keeps increasing as the coordinated PPh_3_ form obtains polarization more rapidly via exchange with free PPh_3_, even as the polarization level of the latter starts to decrease. Therefore, the enhancement of coordinated PPh_3_ under quantitative conditions continues to increase up to 313 K, higher than the turning point for free PPh_3_ (291 K). This behavior can be captured by a simple quantitative exchange model shown in SI (S9 and Figure S9). When the exchange rate is even faster, the shortening of the apparent *T*_1_ of the free PPh_3_ (due to exchange with the bound PPh_3_ in the complex) will limit the polarization build-up time in the elementary OE transfer of polarization from BDPA to the free PPh_3_, which could explain the decrease in the enhancement above 313 K (Figure 1B). In the nonquantitative conditions with significantly shorter recycle delays, this effect is postponed because the loss of polarization is partially compensated for by the shortened *T*_1_ and only a plateau is observed at high temperatures (Figure 1A).

**Figure 3:**
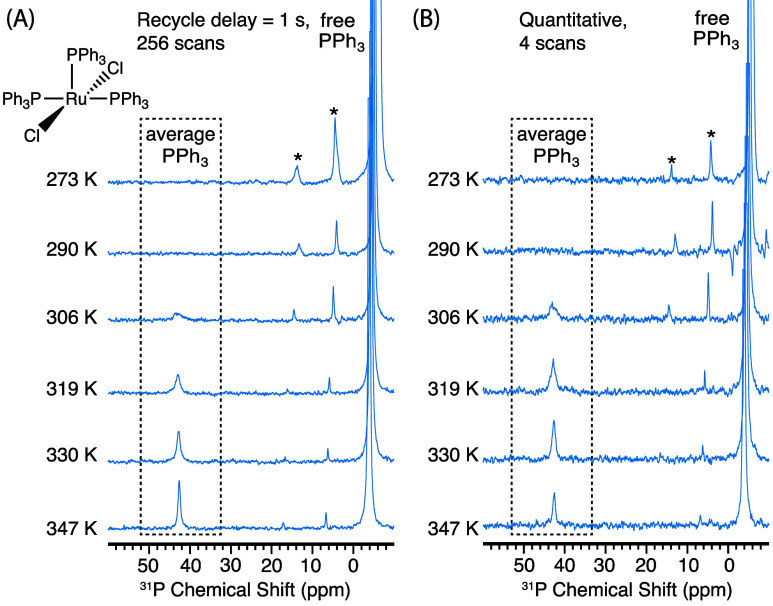
^31^P NMR spectra of ∼4 mM [Ru(PPh_3_)_3_Cl_2_] in benzene-*d*_6_ with 10 mM BDPA used as the PA, and the addition of excess PPh_3_ (100 mM). All NMR experiments were performed at 9.4 T with continuous-wave microwave irradiation at different sample temperatures with (A) recycle delay of 1 s, 256 scans, and (B) recycle delay >5 × *T*_1_ of free PPh_3_ (values shown in Table S4), 4 scans. The spinning sidebands are labeled with asterisks and are due to imperfect shimming. The resonances due to coordinated PPh_3_ are labeled with average PPh_3_.

We next explore the generality of this approach by examining [Ru(PPh_3_)_3_Cl_2_], one of the precursors toward the synthesis of Grubbs-type olefin metathesis catalysts.^43^Figure 3 shows DNP-enhanced ^31^P NMR spectra of the coordinated PPh_3_ ligands in [Ru(PPh_3_)_3_Cl_2_]: alongside the strongly enhanced free PPh_3_ peak at δ ≈ −5 ppm, the coordinated PPh_3_ ligands give an enhanced signal at δ = 43 ppm for temperatures above 300 K. However, in this case, the OE DNP enhancement on the coordinated ligands was not estimated due to the difficulty of obtaining any signals in a microwave-off spectrum (the free ligand is enhanced by a factor 50 here). Nevertheless, we unambiguously confirm that the polarization is transferred from the hyperpolarized free PPh_3_ molecules, as the coordinated PPh_3_ signal disappears when microwave irradiation is applied away from the OE DNP matching conditions. In our setup, this is achieved by sweeping the magnetic field while maintaining all of the other parameters constant (Figure S2B).

It is noted that the coordinated PPh_3_ signal is a singlet, in contrast to the two doublets observed for [Rh(PPh_3_)_3_Cl]. [Ru(PPh_3_)_3_Cl_2_] has a square pyramidal coordination geometry in which the three PPh_3_ ligands occupy two of the four equatorial sites and the remaining axial site, and it is known that the PPh_3_ ligands undergo intramolecular rearrangement at a rate faster than the ^31^P chemical shift differences between the two different sites.^44^ This leads to the observation of only one averaged signal. As the temperature increases, this averaged signal is sharpened due to the accelerated intramolecular site exchange.

The signal intensity of coordinated PPh_3_ shows a similar temperature dependence to that observed for the PPh_3_ ligands in [Rh(PPh_3_)_3_Cl], under both nonquantitative and quantitative conditions (Figure 3). This can be explained by the data shown in Figure 2 (green traces), where both the DNP enhancement under quantitative conditions and the *T*_1_ of free PPh_3_ decrease with temperature in the presence of [Ru(PPh_3_)_3_Cl_2_]. This is again attributed to the effect previously discussed for [Rh(PPh_3_)_3_Cl]. It is worth noting that the decays of both enhancement and *T*_1_ for free PPh_3_ with temperature are less pronounced than those observed in the presence of [Rh(PPh_3_)_3_Cl], indicating an overall weaker loss of polarization effect. In addition, similar to the previous case, the signal intensity of coordinated PPh_3_ reaches a maximum at 330 K, as shown in Figure 3. This turning point occurs at a higher temperature as compared to the [Rh(PPh_3_)_3_Cl] complex (313 K), highlighting again the overall weaker loss of polarization effect in [Ru(PPh_3_)_3_Cl_2_].

**Figure 4:**
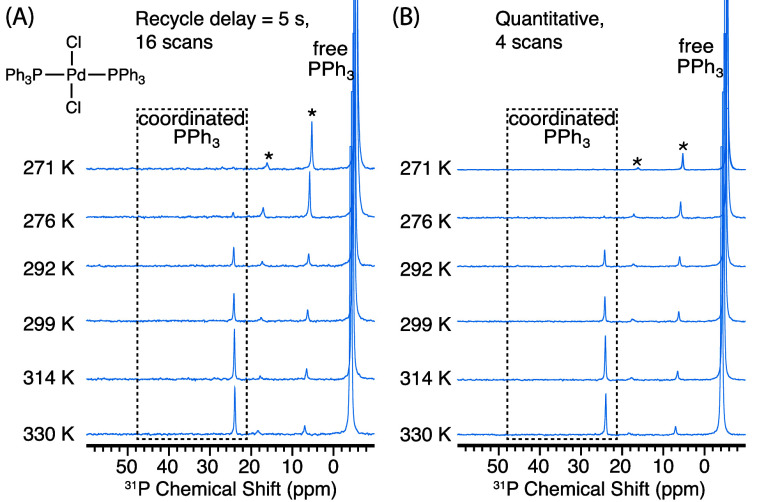
^31^P NMR spectra of ∼3 mM [Pd(PPh_3_)_2_Cl_2_] in benzene-*d*_6_ with 10 mM BDPA used as the PA, and the addition of excess PPh_3_ (100 mM). All NMR experiments were performed at 9.4 T with continuous-wave microwave irradiation at different temperatures with (A) recycle delay of 5 s, 16 scans, and (B) recycle delay >5 × *T*_1_ of free PPh_3_ (values shown in Table S5), 4 scans (except for the spectrum measured at 271 K which is collected by 16 scans with its intensity normalized by 1/4.). The spinning sidebands are labeled with asterisks and are due to imperfect shimming. The resonances due to coordinated PPh_3_ are labeled with coordinated PPh_3_.

Further exploring the generality of the OE DNP protocol, we look at [Pd(PPh_3_)_2_Cl_2_], a frequently used precatalyst for various cross-coupling reactions such as the Suzuki–Miyaura reaction^6^ and the Heck reaction.^5^ As shown in Figure 4, no coordinated PPh_3_ is observed at 271 K, indicating an exchange rate that is too slow for efficient hyperpolarization transfer. However, at higher temperatures, the coordinated PPh_3_ starts to display an enhanced singlet peak at δ = 24 ppm. Similar to the previous observations, the enhancement of the coordinated PPh_3_ ligands arises from the chemical exchange with hyperpolarized free PPh_3_, and no DNP effects are observed in the absence of excess free PPh_3_ or when the field is swept out of the OE DNP matching conditions (Figure S2C). We obtained a signal enhancement at 330 K of 137 ± 24 on the coordinated PPh_3_ (Figure S6B), comparable with the enhancement observed on free PPh_3_ (132 ± 1) under the same condition, indicating complete polarization transfer from the free PPh_3_ to coordinated PPh_3_.

**Figure 5:**
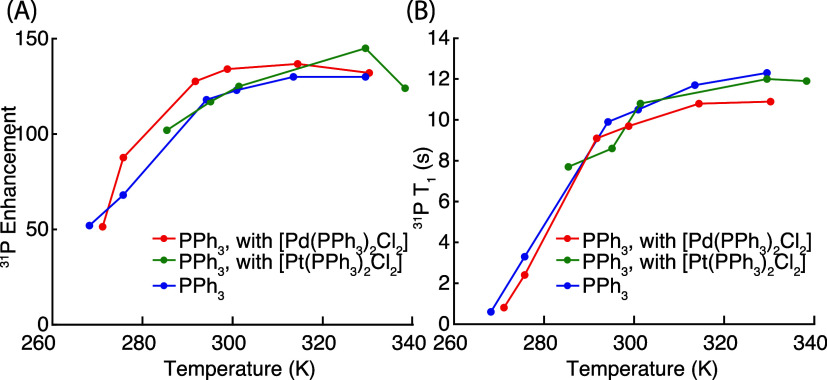
(A) ^31^P DNP enhancement under quantitative NMR conditions and (B) the apparent *T*_1_ measured for the free PPh_3_ signal (100 mM) without metal complexes (blue), with [Pd(PPh_3_)_2_Cl_2_] (red) and [Pt(PPh_3_)_2_Cl_2_] (green), respectively, under microwave irradiation at 9.4 T. In 10 mM BDPA benzene-*d*_6_ solutions.

As opposed to the two examples above, the coordinated PPh_3_ signal recorded under quantitative conditions does not decay at higher temperatures. Rather, it continues to increase until reaching a maximum enhancement of a factor 137 at 330 K, the highest temperature measured for this sample (Figure 4). To understand the reasons behind this difference, we also measured the enhancement under quantitative conditions and the apparent *T*_1_ of free PPh_3_ in the presence of the Pd(II) complex. Data are shown in Figure 5 (red traces). In stark contrast to the trends observed for [Rh(PPh_3_)_3_Cl] and [Ru(PPh_3_)_3_Cl_2_], where the enhancement measured under quantitative conditions and *T*_1_, both decrease at high temperatures (Figure 2), here the enhancement and *T*_1_ both display a monotonic positive temperature dependence. Moreover, the enhancement and *T*_1_ values measured with [Pd(PPh_3_)_2_Cl_2_] are similar to those without metal complexes. The absence of a change in the apparent *T*_1_ of free PPh_3_ suggests that the *T*_1_ of coordinated PPh_3_ in [Pd(PPh_3_)_2_Cl_2_] is not significantly shorter than the free PPh_3_, which is consistent with our measurements shown in Table S7 and the literature.^45,46^ As a result, the loss of polarization effect on the free PPh_3_ that was observed in the two previous examples is negligible for [Pd(PPh_3_)_2_Cl_2_], and the polarization on the free form can therefore be enhanced to its maximum level. As the enhancement of free PPh_3_ is not reduced by the exchange, the fact that coordinated PPh_3_ is more polarized at high temperatures is not surprising. This can also be explained by the quantitative model shown in SI (S9 and Figure S10).

In addition, by using a model for ligand exchange together with the measured ratio of DNP enhancements for the free and coordinated PPh_3_, (as described in detail in the SI), we can determine the activation energy of the ligand exchange reaction for [Pd(PPh_3_)_2_Cl_2_] to be +28 (±10) kcal/mol, as shown in Figure S12, which is consistent with the reported values for the ligand dissociation of Pd(0)-PPh_3_ complexes.^47,48^

**Figure 6:**
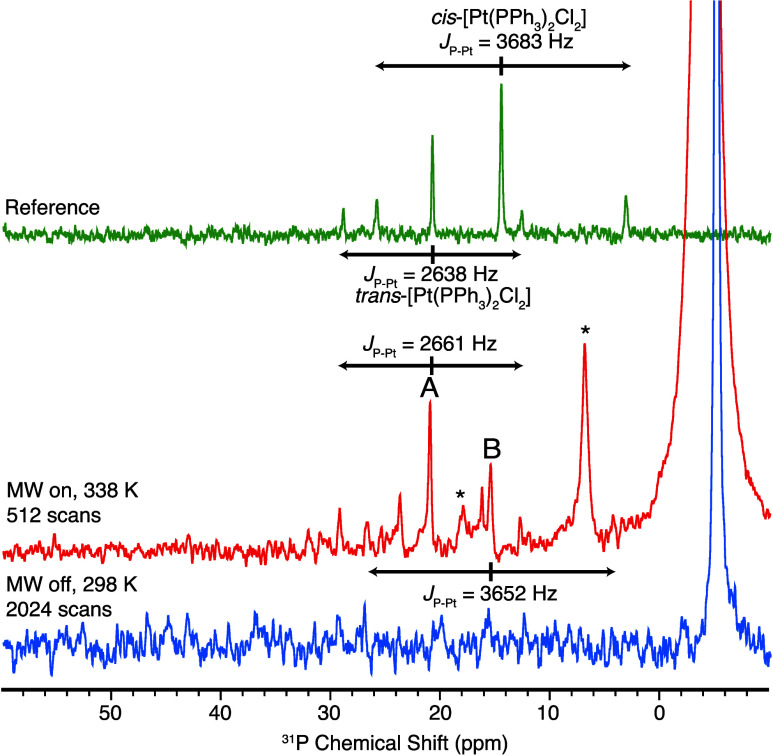
^31^P NMR spectra of saturated [Pt(PPh_3_)_2_Cl_2_] dissolved in dichloromethane (green) and mixed with excess PPh_3_ (100 mM) and 10 mM BDPA in benzene-*d*_6_, obtained at 9.4 T with (red) and without (blue) continuous-wave microwave. The enhanced two peaks are labeled as A and B. The spinning sidebands are labeled with asterisks. The recycle delay is set to 5 s.

Finally, we extended our exploration to [Pt(PPh_3_)_2_Cl_2_]. Unlike [Pd(PPh_3_)_2_Cl_2_], the Pt(II) analogue has a poor solubility in benzene-*d*_6_. Nevertheless, using commercially available *cis*-[Pt(PPh_3_)_2_Cl_2_], we were able to observe the enhanced ^31^P NMR signals of coordinated PPh_3_ using the proposed method, as shown in Figure 6, for an estimated concentration of the complex of only ∼200 μM. Distinct OE DNP-enhanced ^31^P signals for coordinated PPh_3_ are identified, labeled as A and B at δ = 21 and 15 ppm, respectively. These peaks cannot be detected without DNP, as shown in Figure 6 (blue spectrum) and Figure S2D. For both signals A and B, corresponding pairs of satellite peaks are also observed, each with approximately 20% of the intensity of the central peak and separated by 2661 and 3652 Hz for A and B, respectively. These satellite peaks originate from the *J* coupling between ^31^P and ^195^Pt (abundance = 33.7%, spin = 1/2).

When dissolving *cis*-[Pt(PPh_3_)_2_Cl_2_] in dichloromethane, we observed two peaks at δ = 21 and 15 ppm, again with satellites characterized by *J*_P–Pt_ = 2638 and 3683 Hz. These signals correspond to *trans*-[Pt(PPh_3_)_2_Cl_2_] and *cis*-[Pt(PPh_3_)_2_Cl_2_], respectively.^49,50^ Therefore, peaks A and B are likely to be *trans*-[Pt(PPh_3_)_2_Cl_2_] and *cis*-[Pt(PPh_3_)_2_Cl_2_], based on their chemical shift and *J*_P–Pt_. However, it has been reported that in the presence of excess free ligand, bis tertiary phosphine Pt(II) complexes can form the tris tertiary phosphine complexes [Pt(PR_3_)_3_Cl]^+^.^51^ It is also possible that these two peaks, or the other smaller unidentified enhanced peaks, correspond to the ligands in [Pt(PPh_3_)_3_Cl]^+^ (Scheme S1) that might form in our sample.

As shown in Figure 5, the apparent *T*_1_ of free PPh_3_ is not reduced by chemical exchange with bound PPh_3_ at high temperatures; therefore, the loss of polarization effect is negligible. Here, this is likely to occur because of the low concentration of the metal complex. As expected, we observed the maximum enhancement for the coordinated PPh_3_ at 338 K, the highest temperature we could reach with our system (Figure S7). Notably, even though the relative ratio of the free PPh_3_ to the complexes is higher than the Pd case, due to the low concentration of Pt complexes, the signal of the coordinated PPh_3_ increases mainly above 300 K, at a higher temperature than the Pd analogue. We attribute this difference to the slower ligand exchange of the Pt complexes than Pd, which is consistent with previous studies.^52^

## Conclusions

In summary, we have shown here with four illustrative examples of how ligand NMR signals can be enhanced by factors up to 2 orders of magnitude by exchanging relayed hyperpolarization from the free ligands, which was not possible through direct hyperpolarization. The method achieves indirect hyperpolarization of the ligands by combining Overhauser effect DNP with the chemical exchange, provided that the exchange rate is faster than the longitudinal relaxation rate of the free PPh_3_ ligand and slower than the chemical shift difference between the free and coordinated ^31^P resonances. The former requirement can be conveniently fulfilled by adding excess free PPh_3_ ligands in the sample or/and by adjusting the temperature. The excess free PPh_3_ ligands are polarized directly by the polarizing agent, in this work BDPA, and the hyperpolarization to coordinated PPh_3_ ligands is obtained via chemical exchange. Furthermore, we show that the ratio of observed DNP enhancements for the free and coordinated PPh_3_ can be used to determine the activation energy of the ligand exchange reaction.

The broad applicability of this method has been demonstrated through experiments conducted on a family of catalysts that are widely used in organometallic chemistry, including [Rh(PPh_3_)_3_Cl], [Ru(PPh_3_)_3_Cl_2_], [Pd(PPh_3_)_2_Cl_2_], and [Pt(PPh_3_)_2_Cl_2_]. However, we do note that some complexes are incompatible with the current protocol. For example, we have observed that low-valent metal complexes such as Pt(PPh_3_)_4_ reduce the BDPA radical, leading to quenching of the DNP effect. Utilizing a polarizing agent more resistant to such reducing environments could potentially circumvent this issue.^53^

## Experimental Section

To avoid the presence of oxygen in the samples, all solutions were prepared in a glovebox under argon atmosphere. Samples for DNP experiments were prepared by dissolving 10 mM BDPA radical and 100 mM triphenylphosphine (PPh_3_) in benzene-*d*_6_, with or without the metal complex. When present, the metal complexes were set to about 2–4 mM. In case of poor solubility, the complex was first suspended and the solution was then filtered using 0.22 μm syringe filters. For each sample, 10 μL of the solution was transferred into a 3.2 mm sapphire rotor. Kel-f caps were used to seal the rotors to prevent any leakage of the solutions under MAS.

DNP-enhanced experiments were performed on a commercial Bruker Avance III 9.4 T solid-state NMR spectrometer equipped with a low-temperature magic angle spinning (LT-MAS) system and an additional magnetic field sweep coil. Microwave irradiation was performed using a Bruker 4.8 T cryogen-free gyrotron operating at 263 GHz. The magnetic field of the additional sweep coil was set so that the electron paramagnetic resonance (EPR) transition of BDPA was in resonance with the gyrotron microwave frequency. A 3.2 mm LT-MAS DNP probe was used. Samples were spun stably at a MAS rate between 1 and 2 kHz to achieve better sample cooling and narrow down the resonances (compensating for poor sample shimming). The ^31^P NMR spectra were recorded using pulse-acquire experiments. Spectra labeled as quantitative in the main text were acquired with a recycle delay larger than 5 × *T*_1_ of the free PPh_3_^31^P signal.

When the microwave was turned off, variable temperature (VT), bearing, and drive nitrogen gas flows were maintained at room temperature (298 K). The sample temperature at this condition was assumed to be 298 K, and the free PPh_3_ peak at this condition was calibrated to −5 ppm to compensate for the small magnetic field difference (<0.05 mT) introduced by the field sweeping among different experimental sessions. During each session, the magnetic field was stable and the same reference frequency was applied.

Under microwave ON conditions, the microwave-induced heating was compensated by a VT flow at 160–260 K, adjusting the temperature and gas flow rate to regulate the sample temperature. In the MAS probe, the temperatures measured by the thermocouples are different from the real sample temperatures. To estimate the actual sample temperature, the chemical shift of free PPh_3_ was used as an NMR thermometer. To this aim, on a 9.4 T liquid-state NMR machine, we first measured the temperature dependence of the ^31^P chemical shift of PPh_3_ in benzene-*d*_6_ (relative to the chemical shift at 298 K, Figure S1). A linear relation was found (eq S1), which was then used to estimate the sample temperature under MAS in the DNP experiments. In this work, all sample temperatures in the microwave ON experiments are estimated using this method.

## Supporting Information Available

Temperature calibration; additional spectra; tables of relaxation times and intensities; description of the kinetic model; and determination of activation energy; link to the raw data (PDF)
