# Supplementary material for: Probing Homogeneous Catalysts and Precatalysts in Solution by Exchange-Mediated Overhauser Dynamic Nuclear Polarization NMR
Source: J Am Chem Soc. 2024 Apr 30;146(18):12587–94. doi: 10.1021/jacs.4c01570 (PMC11082894; doi:10.1021/jacs.4c01570)
Supplement: Supplementary file 1 — ja4c01570_si_001.pdf [file ja4c01570_si_001.pdf]

Supporting Information for:

Probing homogenous catalysts and pre-catalysts in solution by exchange mediated Overhauser dynamic nuclear polarization NMR

Yu Rao,[1] Federico De Biasi,[1] Ran Wei,[1] Christophe Copéret,[2] Lyndon Emsley[1,]*

[1]Institut des Sciences et Ingénierie Chimiques, Ecole Polytechnique Fédérale de Lausanne (EPFL), CH-1015 Lausanne, Switzerland

[2]Departmentof Chemistry and Applied Biosciences, ETH Zürich, CH-8093 Zürich, Switzerland

Corresponding author:

*lyndon.emsley@epfl.ch

Table of Contents

**Supplementary Information**	**Page**
Raw data statement	S3
S1 Temperature control	S4
S2 DNP OFF experiments	S5
S3 DNP of PPh_3 without metal complexes	S6
S4 $[Rh(PPh_3)_3Cl]$	S7
S5 $[Ru(PPh_3)_3Cl_2]$	S9
S6 $[Pd(PPh_3)_2Cl_2]$	S10
S7 $[Pt(PPh_3)_2Cl_2]$	S11
S8 T_1 measurements	S13
S9 Quantitative model of exchange	S14
S10 Determination of activation energy	S20
References	S22

Raw data statement

The NMR raw data is available (https://doi.org/10.5281/zenodo.11046581) in the original TopSpin, JCAMP formats. Data are made available under the license CC-BY-4.0 (http://creativecommons.org/licenses/by/4.0/ Creative Commons Attribution 4.0 International).

S1 Temperature control

Under microwave heating and cooling gas, the temperature read out by the sensor in the sample stator is not close to the real sample temperature with DNP. Therefore, we use the chemical shift of free PPh_3 as an internal thermometer since it is temperature-dependent. We first measured a sample containing 10 mM BDPA and 40 mM PPh_3 in benzene-d_6 on a liquid-state NMR machine (9.4 T) and found the chemical shift change (relative to 298 K, labelled as Δ) almost linearly with the sample temperature as shown in figure S1 and Table S1. We also tested this dependence using 100 mM PPh_3, 10 mM BDPA and with [Rh(PPh_3)$_3$Cl] and found almost the same relation as listed in Table S1. Therefore, we established a linear relation below to calculate the sample temperature by the relative chemical shift to the experiment at 298 K.

$$T = 42\Delta + 298\ (K)$$

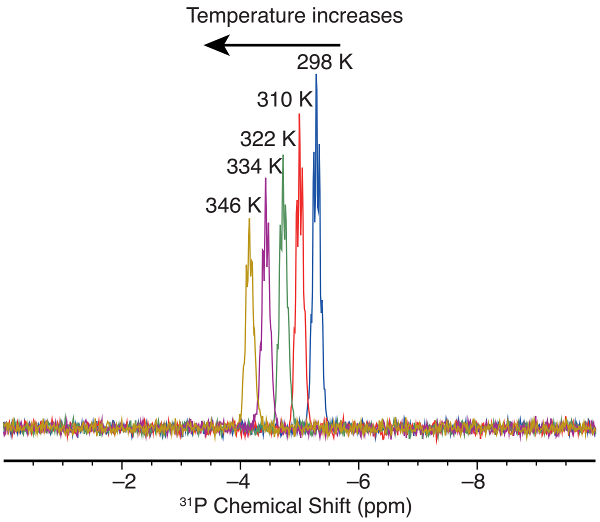

Figure S1. A series of ^{31}P NMR spectra of a benzene-d_6 solution contains 10 mM BDPA and 40 mM PPh_3 measured at different sample temperatures on a 9.4 T liquid NMR spectrometer.

Table S1. The relative chemical shift differences (Δ) of the free PPh_3 measured at different sample temperatures on a routine 400 MHz Bruker liquid-state NMR spectrometer of benzene-d_6 solutions containing BDPA and PPh_3 with and without [Rh(PPh_3)$_3$Cl].

T (K)	40 mM PPh_3, 10 mM BDPA Δ(ppm)	100 mM PPh_3, 10 mM BDPA, 2 mM [Rh(PPh_3)$_3$Cl] Δ(ppm)
298	0	0
310	0.29	0.27
322	0.56	0.56
334	0.85	0.85
346	1.14	1.15

S2 DNP OFF experiments

To rule out the temperature effect which is always coupled with the microwave, we applied a series of microwave experiments but the magnetic field is adjusted so that the electronic spin of BDPA is not on resonance and the DNP effect is therefore quenched. As shown in Figure S2, by comparing these experiments with their corresponding DNP on experiments, it is clear that the coordinated PPh_3 peaks were enhanced because of DNP. Since only the magnetic field was changed by sweeping, we calibrated the free PPh_3 peaks to the same chemical shift as the DNP in the experiment.

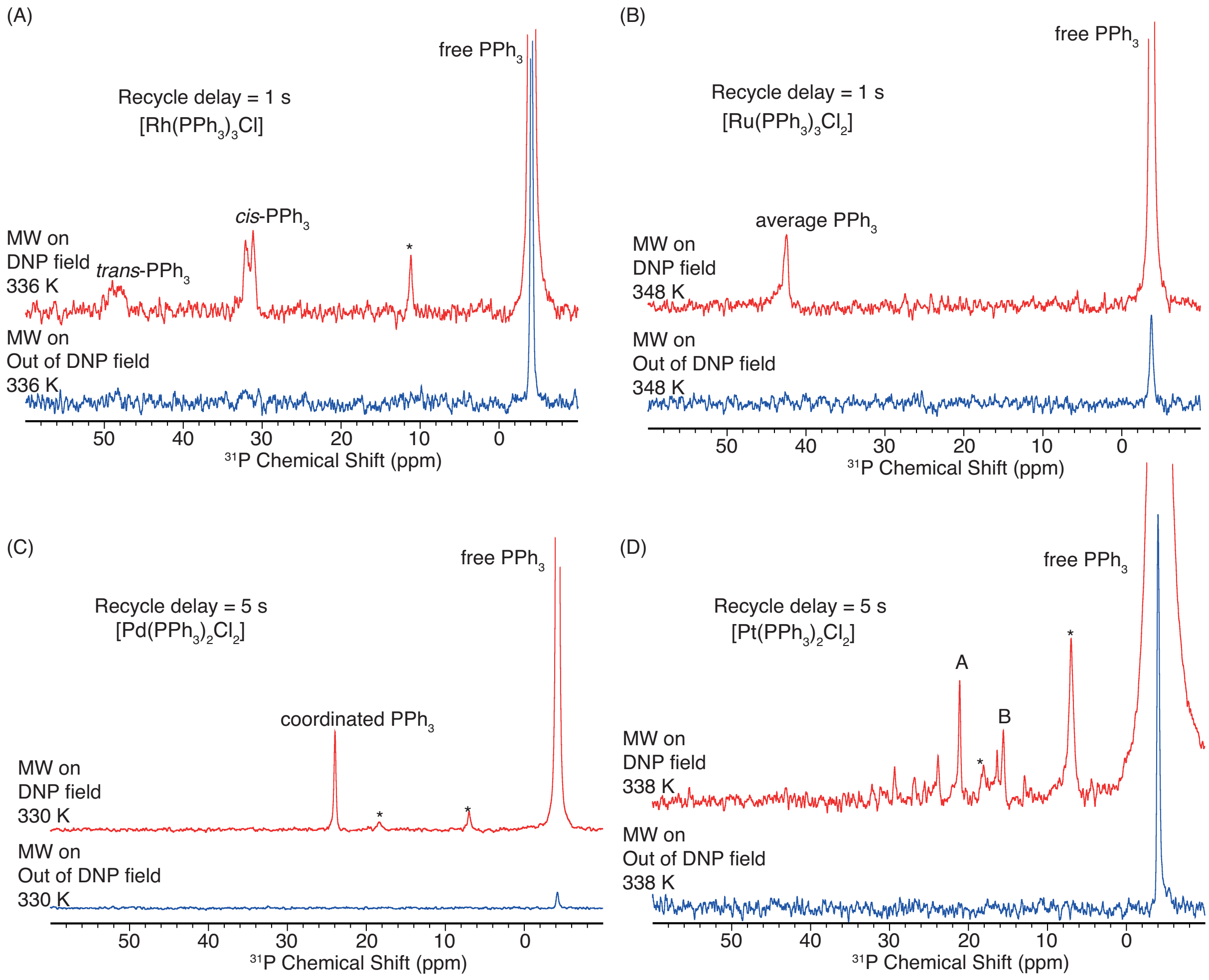

Figure S2. ^{31}P NMR spectra of (A) $[Rh(PPh_3)_3Cl]$, (B) $[Ru(PPh_3)_3Cl_2]$, (C) $[Pd(PPh_3)_2Cl_2]$ and (D) $[Pt(PPh_3)_2Cl_2]$ in benzene-d_6 with the addition of excess PPh_3 (100 mM), 10 mM BDPA obtained at 9.4 T with continuous-wave microwave irradiation at (red) and out of the DNP field (blue). The spinning sidebands are labelled with asterisks. For (A), (B) and (D), spectra in each group were obtained with the same number of scans. While in (C), the DNP off spectrum (blue) is obtained by twice of number of scans of the DNP on spectrum (red), the spectrum intensity is normalized by the number of scans.

S3 DNP of PPh_3 without metal complexes

The DNP experiment of free PPh_3 without any metal complexes has been intensively studied before. Here we perform this experiment to get a reference sample measured with the same set-up for comparison. The resulting DNP enhancement and measured T_1 are plotted in Figure 2 and 5 of the main text with the related conditions and results in Table S2.

Table S2. Summary of the conditions and results of DNP experiment of free PPh_3 without metal complexes with microwave irradiations measured at different temperatures.

VT flow temperature (K)	Δ (ppm)	T (K)	^{31}P T_1 (s)	^{1}H T_1 (s)	ε_P Recycle delay = 5 s	ε_P Quantitative
170	–0.71	268.2	0.6	0.2	132	52
190	–0.53	275.7	3.3	0.9	141	68
210	–0.09	294.2	9.9	2.7	134	118
220	0.07	300.9	10.5	3.0	121	123
240	0.37	313.5	11.7	3.6	119	130
260	0.75	329.5	12.3	3.7	114	130

S4 [Rh(PPh3)3Cl]

For [Rh(PPh_3)$_3$Cl], we also performed the experiment without microwave irradiation at different temperatures and the resulting spectra are shown in Figure S3. It is clear that as the temperature increases, the T_1 of free PPh_3 decreases due to the exchange. In addition, the signal intensity was reduced when acquired with a fixed recycle delay of 1s.

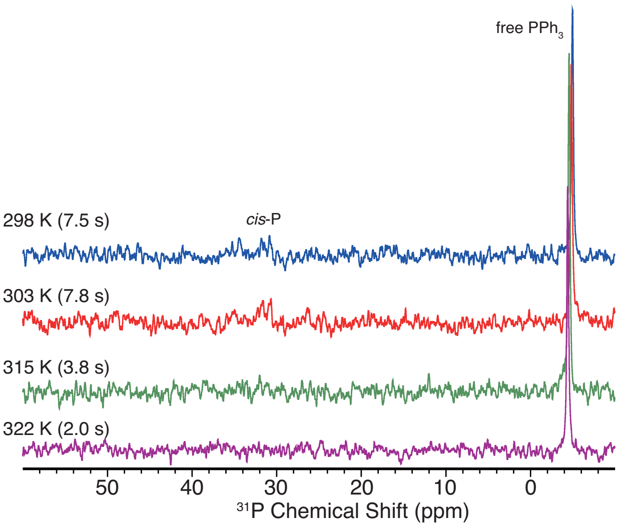

Figure S3. ^{31}P NMR spectra of [Rh(PPh_3)$_3$Cl] mixed with excess PPh_3 (100 mM) and 10 mM BDPA in benzene-d_6, obtained at 9.4 T without continuous-wave microwave at different temperatures. The T_1 of free PPh_3 is shown in the bracket. The recycle delay is set to 1 second and acquired by 1024 scans.

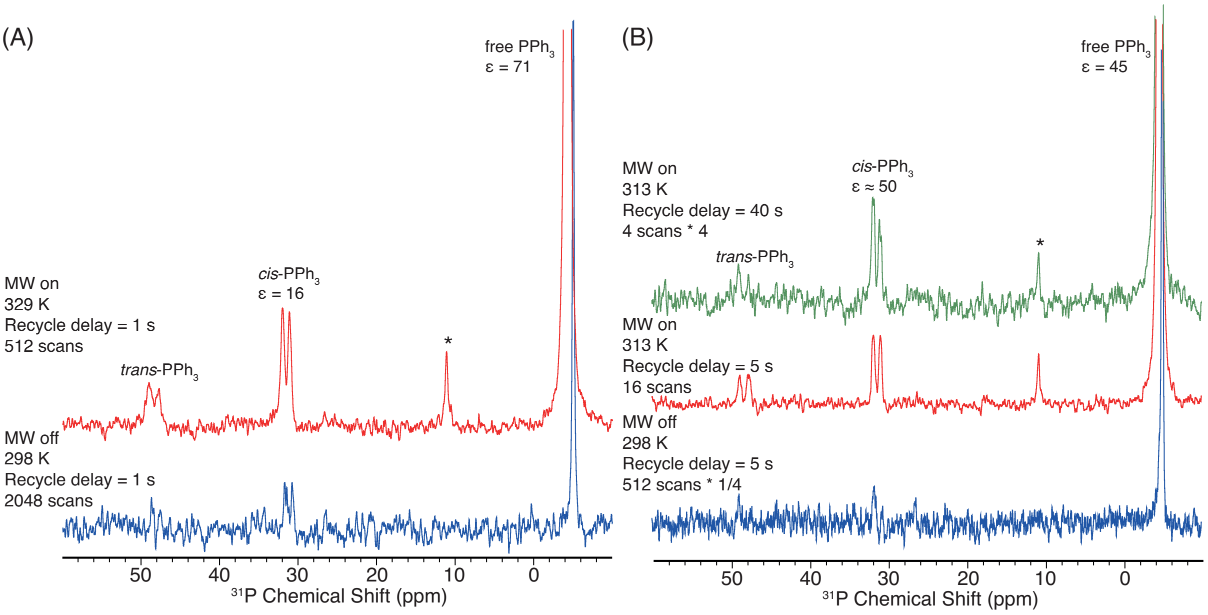

Figure S4. ^{31}P NMR spectra of [Rh(PPh_3)$_3$Cl] mixed with excess PPh_3 (100 mM). 10 mM BDPA in benzene-d_6, obtained at 9.4 T with (red and green) and without (blue) continuous-wave microwave.

Table S3. Summary of the conditions and results of DNP experiment of free PPh_3 with [Rh(PPh_3)$_3$Cl] with microwave irradiations measured at different temperatures (spectra in Figure 1).

VT flow temperature (K)	Δ (ppm)	T (K)	^{31}P T_1 (s)	^{1}H T_1 (s)	ε_P Recycle delay = 1 s		ε_P Quantitative
					Free PPh_3	*Cis*-PPh_3	Free PPh_3
170	–0.82	263.6	0.7	0.2	316	<1	46
190	–0.68	269.4	1.8	0.3	281	<1	77
210	–0.16	291.3	8.0	2.2	116	3	101
220	0.04	299.7	6.5	2.3	114	5	82
240	0.36	313.1	3.9	2.6	93	11	45
260	0.73	328.7	1.3	2.5	71	16	17
270	0.92	336.6	0.8	2.5	58	14	10

S5 [$Ru(PPh_3)_3Cl_2$]

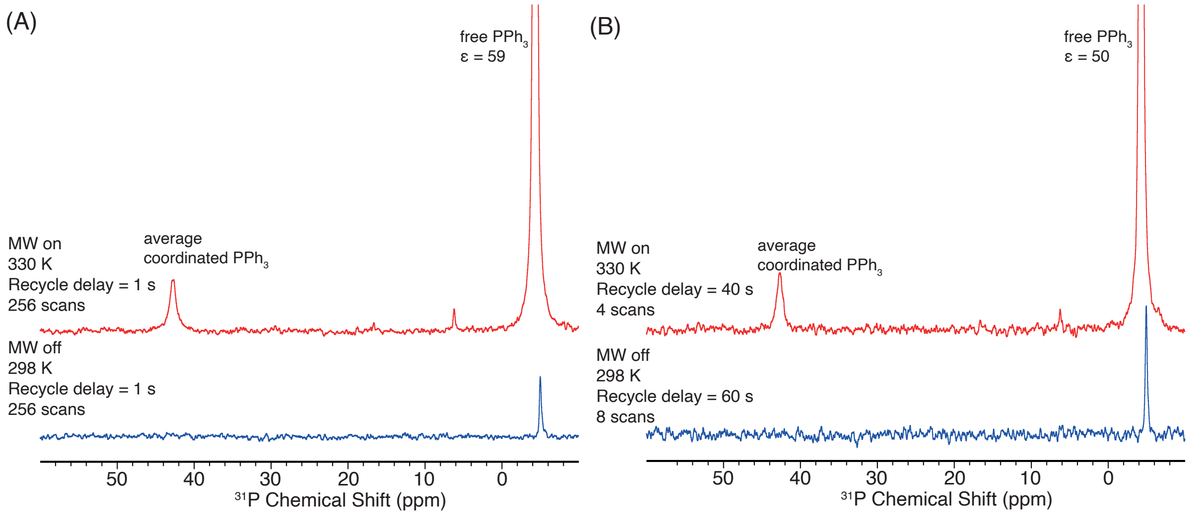

Figure S5. ^{31}P NMR spectra of [$Ru(PPh_3)_3Cl_2$] mixed with excess PPh_3 (100 mM). 10 mM BDPA in benzene-d_6, obtained at 9.4 T with (red) and without (blue) continuous-wave microwave.

Table S4. Summary of the conditions and results of DNP experiment of free PPh_3 with [$Ru(PPh_3)_3Cl_2$] with microwave irradiations measured at different temperatures (spectra in Figure 3).

VT flow temperature (K)	Δ (ppm)	T (K)	^{31}P T_1 (s)	^{1}H T_1 (s)	ε_P Recycle delay = 1 s Free PPh_3	ε_P Quantitative Free PPh_3
180	–0.59	273.2	0.6	0.2	235	53
190	–0.20	289.6	4.4	0.9	89	85
220	0.18	305.6	4.3	1.5	81	75
230	0.50	319.0	4.2	1.6	65	62
230	0.75	329.5	3.8	1.8	59	50
240	1.17	347.1	2.8	2.3	54	32

S6 [Pd(PPh_3)$_2$$Cl_2$]

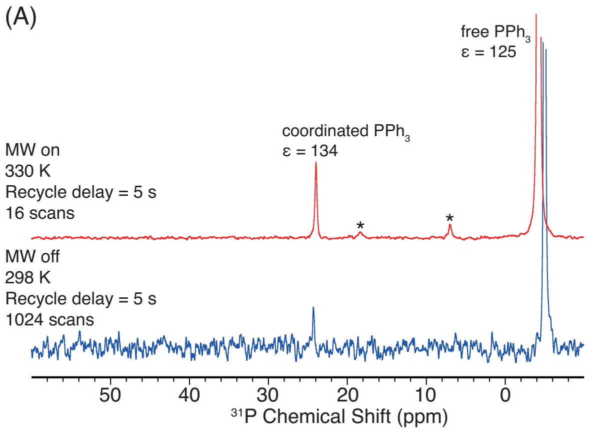

Figure S6. ^{31}P NMR spectra of [Pd(PPh_3)$_2$$Cl_2$] mixed with excess PPh_3 (100 mM). 10 mM BDPA in benzene-d_6, obtained at 9.4 T with (red) and without (blue) continuous-wave microwave.

Table S5. Summary of the conditions and results of DNP experiment of free PPh_3 with [Pd(PPh_3)$_2$$Cl_2$] with microwave irradiations measured at different temperatures (spectra in Figure 4).

VT flow temperature (K)	Δ (ppm)	T (K)	^{31}P T_1 (s)	^{1}H T_1 (s)	ε_P Recycle delay = 5 s		ε_P Quantitative	
					Free PPh_3	Coordinated PPh_3	Free PPh_3	Coordinated PPh_3
160	–0.64	271.1	0.8	0.2	122	7	51	3
190	–0.53	275.7	2.4	0.5	208	16	88	5
210	–0.15	291.7	9.1	1.8	135	50	128	49
220	0.02	298.8	9.7	2.1	131	75	134	75
240	0.39	314.4	10.8	3.2	131	129	137	132
260	0.77	330.3	10.9	4.4	125	134	132	137

S7 [$Pt(PPh_3)_2Cl_2$]

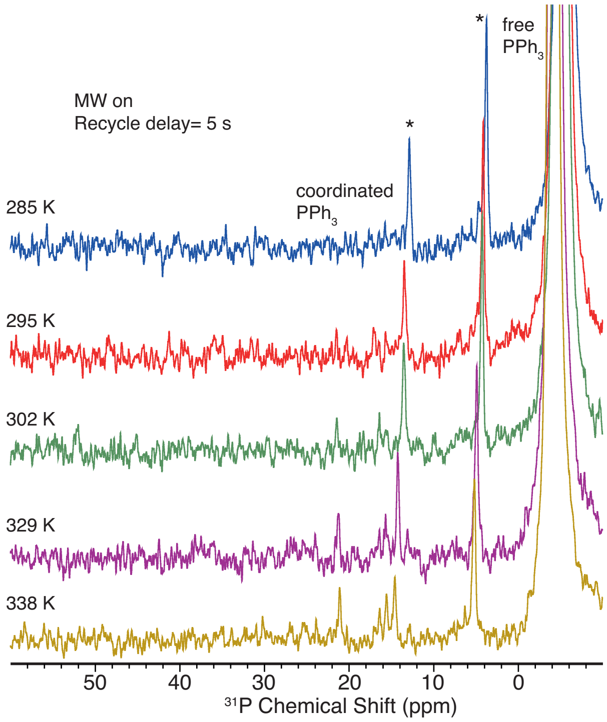

Figure S7. ^{31}P NMR spectra of [$Pt(PPh_3)_2Cl_2$] in benzene-d_6 with the addition of excess PPh_3 (100 mM). 10 mM BDPA was also present in the sample. NMR experiments were performed at 9.4 T with continuous-wave microwave irradiation at different temperatures with a recycle delay of 5 s. The spinning sidebands are labelled with asterisks.

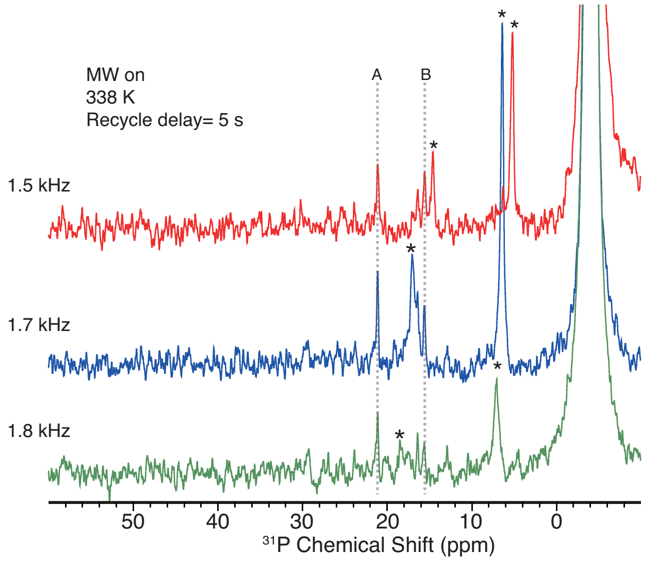

Figure S8. ^{31}P NMR spectra of $[Pt(PPh_3)_2Cl_2]$ in benzene-d_6 with the addition of excess PPh_3 (100 mM). 10 mM BDPA was also present in the sample. NMR experiments were performed at 9.4 T with continuous-wave microwave irradiation at different MAS rates with a recycle delay of 5 s. The spinning sidebands are labelled with asterisks. Two identified enhanced coordinated PPh_3 peaks are labelled with A and B.

Table S6. Summary of the conditions and results of DNP experiment of free PPh_3 with $[Pt(PPh_3)_2Cl_2]$ with microwave irradiations measured at different temperatures (spectra in Figure S7).

VT flow temperature (K)	Δ (ppm)	T (K)	^{31}P T_1 (s)	^{1}H T_1 (s)	ε_P Recycle delay = 5 s Free PPh_3	ε_P Quantitative Free PPh_3
170	–0.30	290.0	7.7	2.0	118	102
200	–0.07	301.8	8.6	2.2	126	117
220	0.08	316.1	10.8	2.2	126	125
240	0.75	330.8	12.0	3.6	117	145
260	0.96	348.0	11.9	4.0	102	124

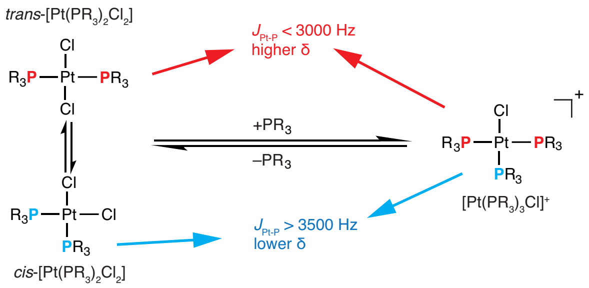

Scheme S1. Formation of a tris tertiary phosphine Pt(II) complex in the presence of excess free phosphine.[1] The phosphorus atoms with chlorine or another phosphorus at the opposite site are labelled in blue or red, respectively.

S8 T_1 measurements

Measuring the intrinsic T_1 for coordinated PPh_3 is difficult because ligand dissociation is always present, which is a problem especially at high temperatures. Here, we measured the T_1 for PPh_3, $[Rh(PPh_3)_3Cl]$ and $[Pd(PPh_3)_2Cl_2]$ in deuterated benzene and the results are shown in Table S7 . It is worth mentioning that some (~10%) free PPh_3 is observed when dissolving pure $[Rh(PPh_3)_3Cl]$ in deuterated benzene. The measured T_1 for coordinated PPh_3 in $[Rh(PPh_3)_3Cl]$ is thus slightly extended by the exchange with the free PPh_3, which has a longer T_1.

Table S7. The T_1 of free PPh_3, $[Rh(PPh_3)_3Cl]$ and $[Pd(PPh_3)_2Cl_2]$ measured in benzene-d_6 at 298 K on a 400 MHz Bruker liquid-state NMR spectrometer.

	PPh_3	$[Rh(PPh_3)_3Cl]$		$[Pd(PPh_3)_2Cl_2]$
		cis-PPh_3	*trans*-PPh_3	
Without BDPA		0.6 s	0.8 s	7.7 s
With ~10 mM BDPA	9.8 s	0.7 s	0.5 s	6.3 s

S9 Quantitative model of exchange

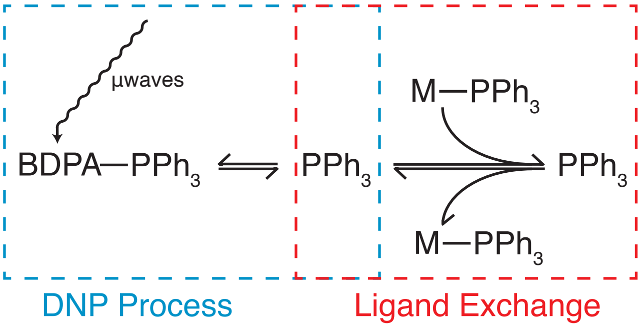

Scheme S2. Schematic representation of the kinetics in the system, which can be divided into the DNP process (blue) and the ligand exchange process (red).

The kinetics in the system with metal complexes could be considered as two parts as shown in Scheme S2. The left part is the DNP process, where the free PPh_3 binds with the BDPA radical in the solution to form a transient complex and the PPh_3 is hyperpolarized during this process. The right part represents the ligand exchange process, where the hyperpolarized free PPh_3 exchanges with a coordinated PPh_3. The DNP process is complex and the BDPA-PPh_3 complex is not observable in the NMR spectrum. To simplify the model, we assume the ligand exchange is relatively slow compared to the elementary DNP process. With this assumption, we can treat the DNP separately by assuming that the magnetization of the free PPh_3 is building up to the DNP enhancement with the intrinsic T_1 (T_{1F} for the free PPh_3). Meanwhile we ignore the weak dipolar-dominated negative DNP effect on the coordinated PPh_3, and then the coordinated PPh_3 should tend to the thermal polarization at the rate $1/T_{1C}$.

$$\frac{dM_F}{dt} = -\frac{M_F - M_{F,DNP}}{T_{1F}}$$

$$\frac{dM_C}{dt} = -\frac{M_C - M_{C,Thermal}}{T_{1C}}$$

Now, we can consider the exchange reaction as presented by Scheme S3.

$$\mathrm{PPh_3 + M{-}PPh_3 \underset{r_-}{\overset{r_+}{\rightleftharpoons}} M{-}PPh_3 + PPh_3}$$

$$\text{Magnetization} \quad M_F \qquad M_C \qquad M_C + n\left(\frac{M_F}{c_F} - \frac{M_C}{c_C}\right) \quad M_F - n\left(\frac{M_F}{c_F} - \frac{M_C}{c_C}\right)$$

Scheme S3. The ligand exchange reaction and the magnetization change after n (mol/L) of exchange reaction.

Here, we assume that the exchange reaction happens instantaneously and the polarization states are preserved after the exchange. Thus, for n mol/L of exchange reaction, it will cause $n\left(\frac{M_F}{c_F} - \frac{M_C}{c_C}\right)$ of magnetization transfer, which is negative for free PPh_3 and positive for coordinated PPh_3. Therefore, there is no net magnetization change in the system due to the exchange reaction at equilibrium. We know that the overall exchange rate r is defined as $r = \frac{dn}{dt}$. In the exchange reaction in Scheme S3, no chemical change is involved and therefore gives an equilibrium constant of 1, and the forward and back transformation rate equals to each other, $r_+=r_-=r$. Combining this exchange effect with the formula above, we have the magnetization change with exchange rate:

$$\frac{dM_F}{dt} = -\frac{M_F - M_{F,DNP}}{T_{1F}} - r\left(\frac{M_F}{c_F} - \frac{M_C}{c_C}\right)$$

$$\frac{dM_C}{dt} = -\frac{M_C - M_{C,Thermal}}{T_{1C}} + r\left(\frac{M_F}{c_F} - \frac{M_C}{c_C}\right)$$

As the concentration of the two species are constant in our experiments, we can use the polarization level to replace the magnetization in the equations above by $M = Pc$ and $\mathrm{d}M = c\,dP$. Then divide the equations by corresponding concentration, and we get

$$\frac{dP_F}{dt} = -\frac{P_F - P_{DNP}}{T_{1F}} - \frac{r(P_F - P_C)}{c_F}$$

$$\frac{dP_C}{dt} = -\frac{P_C - P_{Thermal}}{T_{1C}} + \frac{r(P_F - P_C)}{c_C}$$

The polarization level is proportional to the NMR signal integrals and the scaling factor is the for a given experimental series. Since the enhancement is measured using a microwave off experiment under quantitative conditions, we can assume the polarization at microwave off is the thermal polarization level, and the thermal level is the same for both species.

$$\varepsilon = \frac{I}{I_{off}} = \frac{P}{P_{mw,off}} = \frac{P}{P_{Thermal}}$$

Finally, we can use the enhancement to replace the polarization level by dividing the equations by $P_{thermal}$, and we have:

$$\frac{d\varepsilon_F}{dt} = -\frac{\varepsilon_F - \varepsilon_{DNP}}{T_{1F}} - \frac{r(\varepsilon_F - \varepsilon_C)}{c_F}$$

$$\frac{d\varepsilon_C}{dt} = -\frac{\varepsilon_C - 1}{T_{1C}} + \frac{r(\varepsilon_F - \varepsilon_C)}{c_C}$$

Because we apply saturation pulses before the recycle delay, we have the initial conditions that $\varepsilon_{F,0} = \varepsilon_{C,0} = 0$. The term ε_{DNP} presents the enhancement that the DNP process can achieve in our conditions, since we assume the exchange process does not interfere the DNP process, we use the DNP enhancement measured under similar conditions but without the metal complexes. For [$Rh(PPh_3)_3Cl$], we have ~2 mM complexes that have three coordinated PPh_3 molecules in two different environments with the T_1 of 0.8 s and 0.6 s at room temperature as shown in S8. In principle, we need to treat these two different coordinated PPh_3 separately and need to consider the exchange between these two sites. To simplify, we here treat these two sites as one and the $c_C = 0.006\,M$. The T_1 is in principle temperature dependent, but above room temperature it is relatively stable, so we use $T_{1F} = 10\,s$ and $T_{1C} = 0.8\,s$. ε_{DNP} is also temperature dependent, but stabilizes at about 130 above room temperature when there is no metal complex, so we use ε_{DNP}=130 here.

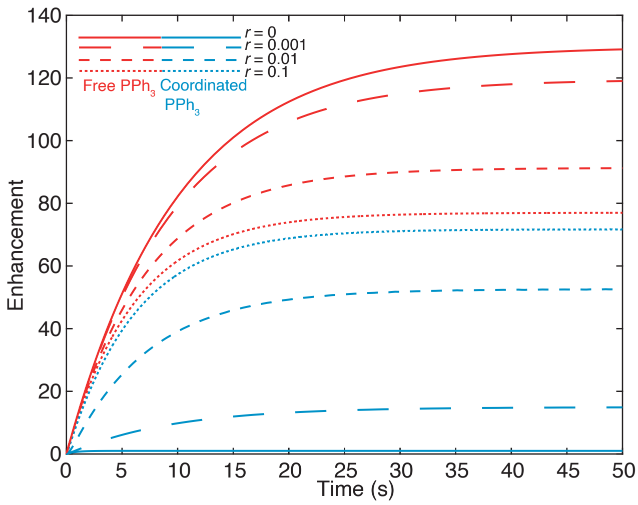

Figure S9. DNP enhancement as a function of polarization time calculated for different exchange rates r (mol L^{-1} s^{-1}), parameters are used according to the experiment with [Rh(PPh3)3Cl] shown in Figure 1. $c_C = 0.006\,M$, $c_F = 0.1\,M$, $T_{1F} = 10\,s$, $T_{1C} = 0.8\,s$ and $\varepsilon_{DNP} = 130$.

With these conditions, we show the predicted change of the enhancement on free and coordinated PPh_3 over 50 s with different exchange rates r in Figure S9. When there is no exchange, the enhancement of free and coordinated PPh_3 at 50 s ($\varepsilon_{F,50s}$ and $\varepsilon_{C,50s}$) is equal to ε_{DNP} and 1, respectively (solid line). As the exchange rate r increases, the enhancement of coordinated PPh_3 increases due to exchange (dashed lines). On the contrary, as explained in the main text, the enhancement of free PPh_3 decreases due to the fast relaxation of the coordinated PPh_3. Meanwhile, the signal build-up time decreases as the exchange rate goes up. These behaviours are consistent with what we observed in Figure 1.

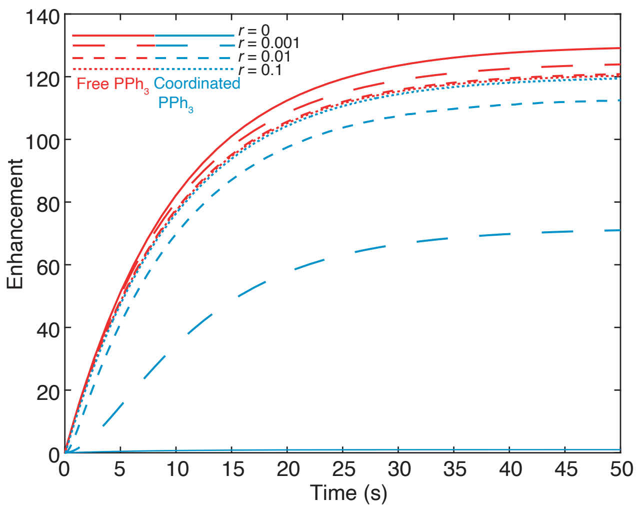

Figure S10. DNP enhancement as a function of polarization time calculated for different exchange rates *r*(mol L^{-1} s^{-1}), parameters are used according to the experiment with [Pd(PPh_3)$_2$$Cl_2$] shown in Figure 4. $c_C = 0.006\ M$, $c_F = 0.1\ M$, $T_{1F} = 10\ s$, $T_{1C} = 8\ s$ and $\varepsilon_{DNP} = 130$.

To demonstrate the behaviour of [Pd(PPh_3)$_2$$Cl_2$] shown in Figure 4, we calculated the enhancements using $T_{1C} = 8\ s$ and found the results shown in Figure S10. As discussed in the main text, the enhancement of the free PPh_3 does not decrease as dramatically as for [Rh(PPh_3)$_3$Cl] because the T_{1C} here is not significantly shorter than the free PPh_3. Consequently, as compare to Figure S9, the coordinated PPh_3 also reaches higher DNP enhancement at the same exchange rate, which is also consistent with our observations.

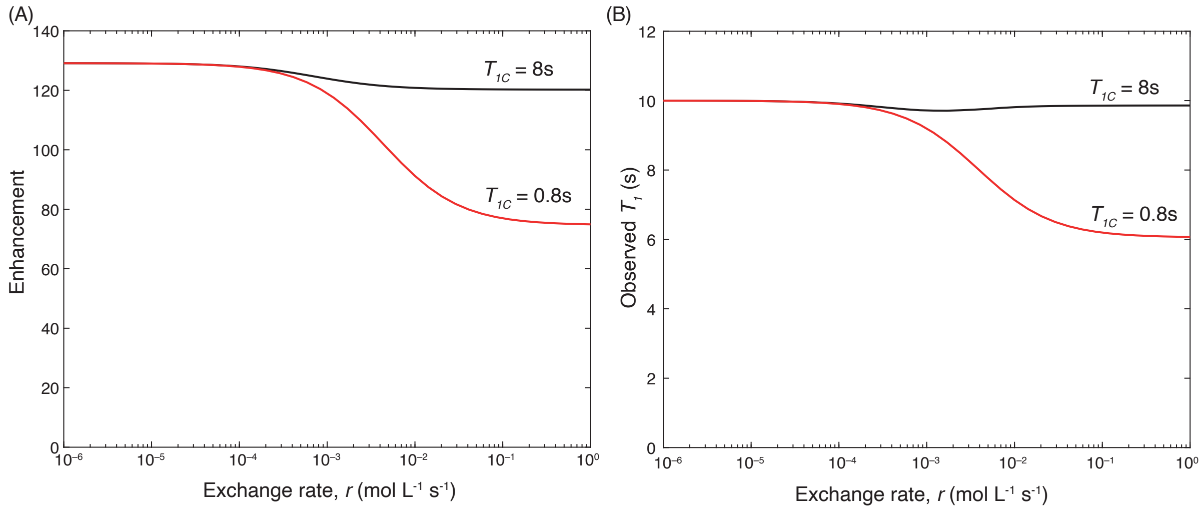

Figure S11. (A) Predicted ^{31}P DNP enhancement at 50s, and (B) the predicted apparent T_1 for the free PPh_3 obtained using the parameters for $[Rh(PPh_3)_3Cl]$ (red) and $[Pd(PPh_3)_2Cl_2]$ (black), respectively. The parameters are given in Figures S9 and S10.

We also fit the predicted enhancement curves for free PPh_3 to extract the predicted apparent T_1. We plot these as a function of exchange rate together with the enhancement of free PPh_3 at 50 s in Figure S11. This strong decay observed on both the enhancement and apparent T_1 is consistent with Figure 2 and 5. The further decay we saw with $[Rh(PPh_3)_3Cl]$ at high exchange rate can not be explained by this simplified model. We believe that this is the regime that the ligand exchange starts to interfere the DNP process, hence our simplified model is no longer valid.

S10 Determination of activation energy

As shown in section S9, the enhancement of the coordinated PPh_3 in a certain regime strongly depends on the exchange rate, which allows us to extract the exchange rate from the DNP experiments. However, the absolute enhancement is related to the ideal DNP enhancement ε_{DNP}, which fluctuates between different experimental sessions due to the hardware performance. The ratio of enhancements, $\frac{\varepsilon_C}{\varepsilon_F}$ is less sensitive to ε_{DNP} especially when the exchange rate is high. Here, we use $\frac{\varepsilon_C}{\varepsilon_F}$ to extract the activation energy of the ligand exchange reaction in $[Pd(PPh_3)_2Cl_2]$ (chosen because of the high signal-to-noise ratio we obtained with this sample and since there is only one environment of coordinated PPh_3).

Table S8. The parameters used to extract the activation energy for the ligand exchange of $[Pd(PPh_3)_2Cl_2]$, experimental data are from Table S5.

T (K)	ε Quantitative		$\frac{\varepsilon_C}{\varepsilon_F}$	Measured ligand exchange rate, ln(r) (mol L^{-1} s^{-1})
	Free PPh_3	Coordinated PPh_3		
275.7	88	5	0.053	-10.2
291.7	128	49	0.38	-7.6
298.8	134	75	0.56	-6.9
314.4	137	132	0.96	-3.8

First, we calculate the ratio of enhancements $\frac{\varepsilon_C}{\varepsilon_F}$ for the data in Table S5, and the results are shown in Table S8. (The data measured at 271.1 K and 330.3 K are not used because their enhancement ratio is too close to 0 and 1 where the ratio is not sensitive to the ligand exchange rate.) We then plot the calculated ratio $\frac{\varepsilon_C}{\varepsilon_F}$ as a function of exchange rate r using the measured parameters from Figure S10, according to the model of section S9, but with ε_{DNP}= 90 and 130 according to the data in Table S8. As Figure S12A shows, ε_{DNP}= 90 and 130 give almost identical curves, with only a small difference observed when the enhancement ratio is close to 0. Therefore, we used the curve with ε_{DNP}= 90 to extract the exchange rates as shown in Figure S12A, and the resulting values are shown in Table S8. Finally, as we assume the reaction rate can be written according to the formula below, the concentrations are constant in this case and can be treated together with the pre-exponential factor.

$$r = k c_F^a c_C^b = A e^{\frac{-E_a}{RT}} c_F^a c_C^b$$

$$ln(r) = \frac{-E_a}{R}\frac{1}{T} + ln(A) + \ln\left(c_F^a c_C^b\right)$$

Finally, we plot the measured rates ln(r) against 1/T and obtain the Arrhenius plot shown in Figure S12B. The fitted slope is 1.4×10^4 ($\pm$ 5×10^3) which corresponds to an activation energy E_a of 28 (± 10) kcal/mol. (If we use only the two points in the linear regime of Figure S12A that potentially have less error on the exchange rates, then the activation energy obtained is 18 kcal/mol.) We note that these activation energies are similar to reported activation energies of the PPh_3 ligand dissociation in palladium or other transition metal complexes (ranging from 15 to 40 kcal/mol)[2-5]. This could suggest that the ligand exchange process is dominated by the dissociation of the PPh_3 ligand.

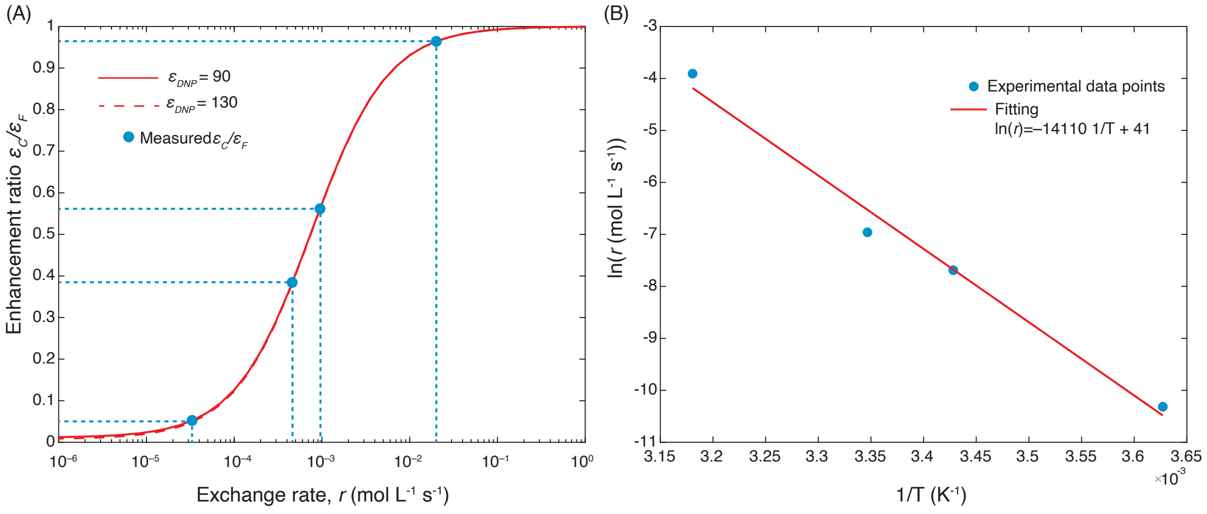

Figure S12. (A) Calculated ratio of enhancements $\frac{\varepsilon_C}{\varepsilon_F}$ at 50 s as a function of exchange rate r (mol L^{-1} s^{-1}) (red lines). Parameters are used according to the experiment with $[Pd(PPh_3)_2Cl_2]$ shown in Figure 4. $c_C = 0.006\,M$, $c_F = 0.1\,M$, $T_{1F} = 10\,s$ $T_{1C} = 8\,s$ and $\varepsilon_{DNP} = 130$ and 90. The blue lines and points indicate the four experimental measured ratios at different temperatures used to determine the rates. (B) Arrhenius plot of the measured rates from (A) (and shown in Table S8), the best fit line is shown in red.

References

(1) Grim, S. O.; Keiter, R. L.; W, M. A Phosphorus-31 Nuclear Magnetic Resonance Study of Tertiary Phosphine Complexes of Platinum(2). *Inorg. Chem.* **1967**, *6* (6), 1133-1137. DOI: 10.1021/ic50052a015.
(2) McMullin, C. L.; Fey, N.; Harvey, J. N. Computed Ligand Effects on the Oxidative Addition of Phenyl Halides to Phosphine Supported Palladium(0) Catalysts. *Dalton Trans.* **2014**, *43* (36), 13545-13556. DOI: 10.1039/c4dt01758g.
(3) Jover, J.; Fey, N.; Purdie, M.; Lloyd-Jones, G. C.; Harvey, J. N. A Computational Study of Phosphine Ligand Effects in Suzuki-Miyaura Coupling. *J. Mol. Catal. A: Chem.* **2010**, *324* (1-2), 39-47. DOI: 10.1016/j.molcata.2010.02.021.
(4) Gamasa, M. P.; Gimeno, J.; GonzalezBernardo, C.; MartinVaca, B. M.; Monti, D.; Bassetti, M. Phosphine Substitution in Indenyl- and Cyclopentadienylruthenium Complexes. Effect of the η^5 Ligand in a Dissociative Pathway. *Organometallics* **1996**, *15* (1), 302-308. DOI: DOI 10.1021/om950428k.
(5) Wovkulich, M. J.; Atwood, J. D. A Trans Effect on the Rate of Ligand Dissociation from Octahedral Organo-Metallic Complexes - Dissociation of L' from Cr(Co)4LL' (L, L' = PBu3, PPh_3, $P(OPh)_3$, $P(OMe)_3$, $AsPh_3$). *Organometallics* **1982**, *1* (10), 1316-1321. DOI: DOI 10.1021/om00070a012.
